# Assessment of Step Tracker Mobile Applications for the Promotion of Physical Activity by Adolescents Based on Their Weight Status

**DOI:** 10.1155/2024/8038334

**Published:** 2024-09-30

**Authors:** Cristina M. Ponce-Ramírez, Adrián Mateo-Orcajada, Lucía Abenza-Cano, Raquel Vaquero-Cristóbal

**Affiliations:** ^1^ Facultad de Deporte UCAM Universidad Católica de Murcia, Murcia 30107, Spain; ^2^ Research Group Movement Sciences and Sport (MS&SPORT) Department of Physical Activity and Sport Faculty of Sport Sciences University of Murcia, Murcia, Spain

## Abstract

The objectives of the present study were to analyze the adherence of normal weight and overweight/obese adolescents to intervention with mobile applications; to establish the differences in the evaluation of the mobile application and in the problematic use of the mobile phone between normal weight and overweight/obese adolescents; and to determine the relationship between the distance travelled, the evaluation of the mobile applications, and the conflictive use of the mobile phone. A quasi-experimental design was carried out with the participation of 70 adolescents between 12 and 16 years old (mean age: 14.25 ± 1.23 years old; 38 normal weight and 32 overweight/obese). The adolescents completed a 10-week intervention in which they used step tracker mobile applications for the promotion of physical activity, a minimum of three times per week. Problematic mobile phone use and adolescent ratings of the application used were measured. The results showed no significant differences in adolescents' adherence to the intervention according to the mobile application used (*p* = 0.191) or weight status (*p* = 0.202). In addition, significant differences were not found in the assessment of mobile applications within the group of overweight and obese adolescents: engagement (*p* = 0.471), functionality (*p* = 0.319), aesthetics (*p* = 0.378), information (*p* = 0.184), usability (*p* = 0.154), or perceived impact (*p* = 0.139), although differences were found in the assessment made by normal-weight adolescents in information (*p* = 0.029), usability (*p* = 0.029), and perceived impact (*p* = 0.044), where Pacer had better scores than Pokémon Go in the first two dimensions. No greater problematic mobile phone use was found after the intervention according to weight status (*p* = 0.311) nor the mobile application used (*p* = 0.985). It can be concluded that there is similar adherence among normal weight and overweight/obese adolescents to interventions with mobile applications to promote physical activity. It is noteworthy that adolescents, regardless of weight status, showed a positive perception towards the use of these mobile applications.

## 1. Introduction

The prevalence of overweightness and obesity has increased exponentially in recent decades in the child and adolescent population [1]. This increase has been so acute that the World Health Organization (WHO) indicates that 24.9% of adolescents between 10 and 19 years of age are overweight, while 7.1% of adolescents in this age group are obese [2]. Spain is among the countries with the highest incidence of these conditions, with almost one million children and adolescents aged 2–17 years being obese and more than two million being overweight [3].

In an attempt to reverse this situation, previous research has shown the effectiveness of promoting certain healthy lifestyle habits in the overweight/obese adolescent population [4], including the practice of physical activity [5]. Thus, recent studies have carried out physical activity interventions for the overweight/obese adolescent population in school and out-of-school settings, as well as for subjects at risk of suffering from these pathologies, finding beneficial effects on the health of this population [6–8]. However, most research shows limited effectiveness [9], as this population shows a high resistance to physical activity interventions, being considered as hard to reach [5].

However, physical activity interventions that include technological devices such as mobile apps could provide a great opportunity for overweight/obese adolescents [5, 10]. Indeed, interventions using such devices with overweight/obese adolescents have found an increase in physical activity when using electronic devices [11], with associated health benefits [5]. However, after the first few weeks of using these devices, a significant decrease in adherence to the intervention has been observed, which could be due to the loss of novelty [12], with the consequent loss of effectiveness of this type of intervention [13].

However, an issue to take into consideration when using mobile applications to promote healthy habits among adolescents is that this type of intervention is usually also associated with an increase in the number of hours of use of other types of mobile applications [14]. This is especially important when considering that the adolescent population is particularly sensitive to the problem of overuse of technological devices [15, 16]. This situation is even more alarming among overweight/obese adolescents [17]. This is because overweight and obese adolescents, those with a subjective perception of being overweight, and those with inappropriate weight control behaviour have higher rates of problematic mobile phone use [18]. In addition, this problematic mobile phone use is associated with unhealthy behaviours such as inactivity and sedentary lifestyles [19, 20], unhealthy food consumption [21, 22], and abnormal sleep patterns [20], all of which are linked to overweight and obesity. In addition, mobile phone use alone is associated with increased accumulation of body fat [23], and adolescents with problematic mobile phones are two times more likely than their peers to fall within an overweight/obese body mass index(BMI) status [19, 24].

Given the previous questions, it would be advisable to know the opinion of, and assessment by, adolescents in general and overweight/obese adolescents in particular, mobile applications aimed at physical activity, in order to optimize their use and the effectiveness of interventions based on them [7, 25]. This need has been identified in previous research which has shown that currently designed diet, physical activity, and sedentary behaviour apps are of moderate quality, scoring high on functionality, and including numerous techniques for behaviour change [26]. However, no previous study has investigated how overweight/obese adolescents rate the mobile applications that promote physical activity, even though this population might have different requirements than normal-weight adolescents [26]. Therefore, this is an area of study that is completely unknown in previous scientific literature and needs to be addressed, as previous studies have shown that adolescents' evaluation of the apps they use could be a determining factor in the time they spend using them [27, 28]. In view of all that has been mentioned so far, no previous research has analyzed adolescents' assessments of step tracker mobile apps, nor has it examined whether there might be differences in the assessment given to this type of app by adolescents depending on their weight status. For these reasons, the objectives of this research were (a) to analyze the adherence of normal weight and overweight/obese adolescents to intervention with mobile apps in out-of-school hours promoted from the school subject of physical education, (b) to determine the differences in the objective and subjective assessment of mobile apps according to weight status and the mobile app used, (c) to determine the differences in the problematic use of mobile phones according to weight status and the mobile application used by adolescents, and (d) to establish the relationship between distance travelled with mobile application use, ratings, and problematic mobile phone use.

In view of the objectives of this research, as well as the lack of previous research in this field, the following research hypotheses are posed: overweight adolescents will have lower adherence to the intervention with mobile physical activity applications (H1); there will be differences in the assessment of the applications according to weight status and the app used, and gamified applications will lead to more adherence than nongamified ones; there will be differences in the app rating according to weight status and the app used (H2); problematic mobile phone use will increase after the intervention period (H3); and there will be a relationship between the distance travelled with the app, the app rating, and problematic mobile phone use (H4).

## 2. Materials and Methods

### 2.1. Design

A quasiexperimental design was carried out with two experimental groups, the first being composed of normal-weight adolescents and the second of overweight/obese adolescents. All participants used a mobile step tracker application (Strava, Pacer, Pokémon Go, or Map My Walk) for 10 weeks, at least three times a week and walking a certain distance.

Before the start of the intervention (pretest), participants completed the Questionnaire of Experiences Related to Mobile Phones (CERM). This was performed during physical education class time and in a classroom where the students could complete the questionnaire without noise or external factors that could distract them. Once the pretest had been completed, the 10-week intervention with mobile applications began, in which a researcher was in charge of recording the daily steps taken by each adolescent. At the end of the intervention (posttest), the students completed the CERM questionnaire and the “User Version of the Mobile Application Rating Scale” (uMARS), again using physical education class time and a classroom isolated from noise. The researchers had no influence on the students' responses to the questionnaires and were only responsible for resolving any possible doubts, if any.

The Consolidated Standards of Reporting Trials (CONSORTs) guidelines were followed for the research design, and the study was registered prior to commencement at ClinicalTrials.gov (code: NCT06089876). The institutional ethics committee of the Catholic University of Murcia approved the study design according to the World Medical Association and following the Declaration of Helsinki (code: CE022102).

### 2.2. Participants

For the present research, a school was selected in the Region of Murcia with the largest number of adolescents enrolled in compulsory secondary education (CSE) in its municipality. Once the school had agreed to participate, the school's management team was contacted, as well as those responsible for the physical education department. Subsequently, an informative meeting was held with the adolescents and their parents to explain the purpose and procedure of the study in detail. The adolescents who expressed their willingness to participate provided informed consent signed by them and their parents.

The sampling was non-probabilistic by convenience, through the selection of adolescents with a normal weight (BMI: 18.5–24.9) and overweight/obese (BMI: >25) available at the selected school. The sample size was calculated using the standard deviation (SD) of the physical activity variable after an intervention with mobile apps in adolescents with obesity (SD = 3.7) [20]. Thus, the minimum sample required for each of the intervention groups was 16 subjects, with a confidence interval (CI) of 95% and an error (*d*) of 1.62. The statistical program used to calculate the sample size was Rstudio 3.15.0 (Rstudio Inc., United States).

**Figure 1:**
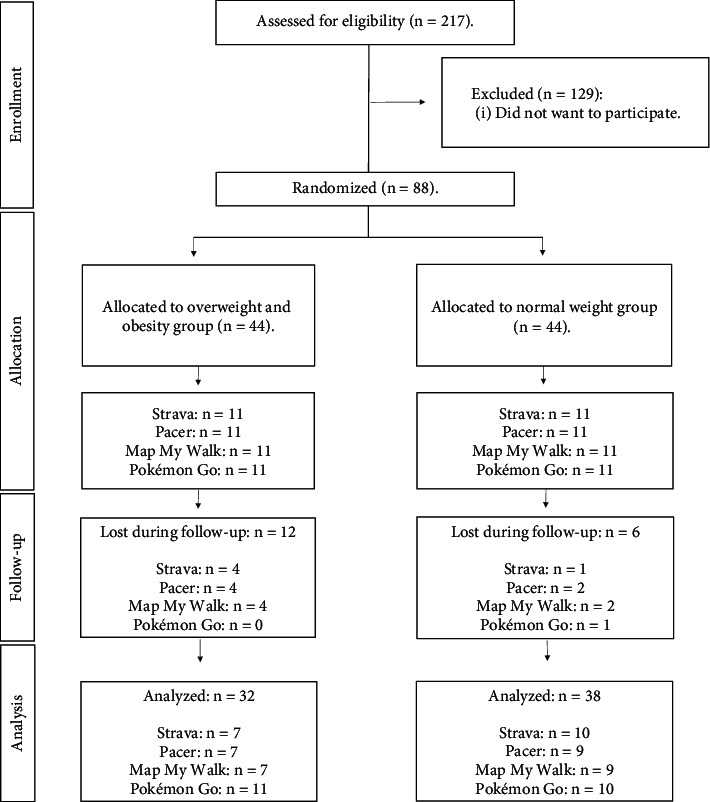
Sample selection flowchart.

A total of 88 CSE adolescents initially participated in the research (42 males and 46 females; 44 normal weight and 44 overweight/obese; 22 on Strava, 22 on Pacer, 22 on Pokémon Go, and 22 on Map My Walk), aged between 12 and 16 years (mean age: 14.25 ± 1.23 years old). Although the sample size is small, it is similar to that of most previous research using physical activity programs in overweight/obese adolescents [29, 30]. Furthermore, the division into mobile app subgroups is necessary because previous research has shown the importance of comparing different mobile apps in adolescents, as their interface and features may lead to different results [26, 31]. The inclusion criteria were (a) aged between 12 and 16 years old and (b) attending CSE. The exclusion criteria were (a) not owning a mobile phone and (b) not attending the scheduled measurement sessions. The final participation was 70 adolescents (31 males and 39 females; 38 normal weight and 32 overweight/obese; 17 on Strava, 16 on Pacer, 21 on Pokémon Go, and 16 on Map My Walk). A total of 18 adolescents dropped out of the study between the normal-weight (*n* = 6) and overweight/obese (*n* = 12) groups. The reasons were as follows: they did not want to continue in the research (normal weight: *n* = 3; overweight/obesity: *n* = 4), they changed schools (normal weight: *n* = 1; overweight/obesity: *n* = 3), and they did not attend the posttest measurements (normal weight: *n* = 2; overweight/obesity: *n* = 5). The sample selection flowchart is shown in Figure 1.

### 2.3. Mobile App Interventions

Participants in both experimental groups were randomly assigned to one of the selected mobile apps (Strava, Pacer, Map My Walk, and Pokémon Go). Randomization was carried out by the principal investigator using a computer-generated random number table in the presence of other researchers not involved in the study. This divided the adolescents into two groups (normal weight and overweight/obese) and into each of the four mobile app groups. No gender randomization was performed because the number of males and females in both groups was homogeneous. Baseline measurements were taken after the randomization process. The researchers were blinded to the mobile application used by the adolescents during the pre- and posttest measurements.

The apps were chosen because they were similar to each other and useful for promoting physical activity, according to previous research [17], and also for including various techniques for behavioural change [28]. Strava, Pacer, and Map My Walk are considered pedometers that include messages and reminders to promote physical activity, while Pokémon Go is a video game that promotes physical activity through the Pokémon that appear in the game as you walk through different areas, as well as the rewards it includes the longer you walk [32].

Before starting the intervention, an explanation was provided to the adolescents in each of the intervention groups about how to use the mobile application, with emphasis on how to activate the application each day of the week they used it, as well as where to observe the steps taken on the application's interface. The adolescents were also informed that completing the intervention would raise their final grade in the physical education subject by 10%.

After this, the adolescents used the mobile physical activity apps for 10 weeks outside school hours. The proposed target for the first week was 4.5 km each day they used the app, which corresponds to 7152 steps [33], following the recommendations from previous research [34]. The distance to be covered was progressively increased each week at a rate of 595 steps/week, ending with a total of 12,520 steps on the 10th week, each time they used the application, which corresponds to 8 km, the minimum distance recommended to reach a moderate to vigorous intensity of physical activity [35].

### 2.4. Instruments

Problematic mobile phone use was assessed using the CERM questionnaire. This questionnaire is composed of 10 items that are completed with a Likert scale ranging from 1 to 4 points (1: *never*; 4: *almost always*), and the sum of the 10 items provides the final score of the questionnaire [36]. In addition, this questionnaire provides two dimensions, each composed of five items: conflictive use and emotional use. Conflictive use refers to the negative situations in which users may be involved due to inappropriate use of the mobile phone in relation to lost opportunities, work/academic performance, addiction, and sleep disturbance, while emotional use refers to the need to use the mobile phone and its influence on psychological variables such as state of restlessness, sadness, anger, or irritation [36]. The questionnaire has a high internal consistency for the final score (*α* = 0.80), as well as for both dimensions (conflictive use: *α* = 0.81; emotional use: *α* = 0.75) [36].

The mobile apps were rated by the adolescents by using the uMARS [37]. This scale is composed of 26 items that allow rating the quality of apps objectively and subjectively. The original version of it has excellent internal consistency (*α* = 0.90) [37], which is maintained in the Spanish version (*α* = 0.89) [38]. The objective part of the questionnaire is completed using a Likert scale ranging from 1 to 5 points (1, *inadequate*; 5, *excellent*) that allows the evaluation of four dimensions: engagement (*α* = 0.80), functionality (*α* = 0.70), aesthetics (*α* = 0.71), and information (*α* = 0.78) [37]. Engagement refers to the ability of the application to be fun, interesting, customizable, interactive, and has prompts (sends alerts, messages, reminders, and feedback and enables sharing); functionality refers to the app functioning, easy to learn, navigation, flow logic, and gestural design of the app; aesthetics refers to the graphic design, overall visual appeal, color scheme, and stylistic consistency; information indicates that it contains high-quality information (text, feedback, measures, and references) from a credible source [37]. The subjective part includes 10 questions assessed using a Likert scale ranging from 1 to 5 points, in which usability and perceived impact are evaluated [37]. Usability refers to the potential for future use of the application, as well as the possibility of recommending it to other users, while perceived impact measures awareness, knowledge, attitudes, intention to change, help-seeking, and behavioural change brought about by the use of the app [37].

### 2.5. Data Analysis

The normality of the data was analyzed using the Kolmogorov–Smirnov test, skewness, and kurtosis. Since the variables followed a normal distribution, parametric tests were used for analysis. A chi-square test (*χ*^2^) was performed to analyze differences in adherence and dropout rate of mobile app use according to adolescents' weight status. The corrected standardized residuals were used to determine significance, establishing the value of ±1.96 as the reference value. Cramer's *V* was used for the post hoc comparison of the 2 × 2 tables, and the contingency coefficient was used in the 2 × *n* tables to obtain the statistical value. The maximum expected value was 0.707; *r* < 0.3 indicated a low association; *r* < 0.5 indicated a moderate association; and *r* > 0.5 indicated a high association [39]. Two ANOVAs were carried out, the first being useful to analyze differences in app ratings between normal weight and overweight/obese adolescents, while the second was used to analyze differences between the different mobile applications in the normal weight and overweight/obese groups. A Bonferroni post hoc analysis was used to determine between which mobile apps the differences were found. A MANCOVA was performed to establish differences in problematic mobile phone use as a function of the mobile application used and weight status, according to the covariate “quality of the app.” Subsequently, a Pearson correlation (*r*) analysis was carried out to establish the relationship between the distances travelled using the mobile app and the app rating. In this case, the correlation was defined as strong: *r* ≥ 0.5; moderate: *r* ≥ 0.3; weak: *r* > 0; or nonexistent correlation: *r* = 0. Partial eta squared (*η*^2^) was used to calculate the effect size (ES) and was defined as small: ES ≥ 0.10; moderate: ES ≥ 0.30; large: ES ≥ 1.2; or very large: ES ≥ 2.0, with an error of *p* < 0.05 [40]. A value of *p* < 0.05 was set to determine statistical significance. The statistical analysis was performed using the SPSS statistical package (v.25.0; SPSS Inc., IL).

## 3. Results

Table 1 shows the differences in adolescents' adherence to the intervention according to the mobile application used and weight status. The results did not show significant differences between the mobile applications used (*p* = 0.191) or when comparing normal-weight adolescents with overweight/obese adolescents (*p* = 0.202).

The intergroup differences in the rating of each of the apps between normal weight and overweight/obese adolescents are presented in Table 2. The results did not show significant differences between normal weight and overweight/obese adolescents in the rating of the different mobile apps in any of the dimensions (engagement: *p* = 0.471–0.941; functionality: *p* = 0.319–0.905; aesthetics: *p* = 0.378–0.890; information: *p* = 0.184–0.995; usability; *p* = 0.154–0.854; perceived impact: *p* = 0.139–0.794).

Table 3 presents the differences in the rating of the different mobile applications used in the normal weight group and also in the overweight/obese group (intragroup differences). The results showed significant differences in the information (*p* = 0.029), usability (*p* = 0.029), and perceived impact (*p* = 0.044) dimensions in the group of normal-weight adolescents.  Table 4 presents the Bonferroni post hoc analysis, which shows that in normal-weight adolescents, Pokémon Go obtained higher scores than Pacer in the information (*p* = 0.043), usability (*p* = 0.046), and perceived impact (*p* = 0.045) dimensions.

The analysis of problematic mobile phone use is presented in Table 5. No significant changes in problematic mobile phone use were observed as a function of weight status and mobile application used (*p* = 0.311–0.985). The inclusion of the covariate objective assessment of the app through the uMARS questionnaire was also not shown to significantly influence any of the variables analyzed (*p* = 0.311–0.985).

Table 6 shows the correlation analysis of the study variables in the normal weight and overweight/obese adolescents. The results show that in normal-weight adolescents, a higher training volume was positively related to a higher conflictive use of mobile phones (*p* = 0.005). However, in overweight adolescents, no higher conflictive use of the mobile phone was observed (*p* = 0.971), while the results also showed that a higher training volume was negatively correlated with engagement (*p* = 0.009), functionality (*p* = 0.003), aesthetics (*p* = 0.006), information (*p* = 0.017), usability (*p* = 0.025), and perceived impact (*p* = 0.006).

## 4. Discussion

The first objective of the present investigation was to analyze the adherence of normal weight and overweight/obese adolescents to an intervention with mobile apps during out-of-school hours promoted by the school subject of physical education. A relevant finding of the present investigation was that there was no significant difference in the dropout rate during the intervention as a function of weight status. These results differ from those found in previous research, which labeled overweight/obese adolescents as a “hard-to-reach population” due to their difficulty in initiating and adhering to physical exercise programs [5]. However, in this study, we did not find such reluctance to practice physical exercise in the overweight and obese adolescent population, which could be due to the fact that they opted for a reward in the form of a 10% increase in their final grade of the physical education course, which could have favored extrinsic motivation as the main factor for following the intervention [41, 42]. In this regard, previous research has shown that maintaining high levels of motivation towards physical activity is associated with higher levels of practice and retention in adolescents with obesity [43, 44] and that rewards can contribute to increased extrinsic motivation and participation in physical activity programs [45–47].

Another possible explanation for the absence of differences in dropout rates as a function of weight status in the present investigation is that most of the programs previously used for the promotion of physical exercise in overweight and obese populations have promoted exercise at moderate or vigorous intensity in order to maximize their effects on body composition [48, 49] and physical condition [25, 50]. However, the exercise performers' perceptions and feelings at these intensities may not be pleasurable, causing many to drop out [51]. In contrast, the present study presented an intervention program that could be performed at lower intensities, which could have favored the lack of differences in the dropout rate between overweight and obese participants and normal-weight participants, as previous research has shown that low-intensity training protocols can generate greater adherence [52, 53].

As a third argument, the scientific literature states that mobile applications could be a good resource to motivate overweight/obese adolescents to engage in physical activity, as they are an attractive resource for them [54, 55], especially when presented in a novel way [56]. Therefore, it is possible that the current intervention, which presented adolescents with a motivating and novel way of promoting physical activity, has succeeded in overcoming the barriers they have against physical activity [56].

Another significant result of the present research was that the abandonment rates according to the different mobile applications used were similar. The results contrast with those presented in previous research, in which it was found that mobile applications, when gamified, could have a lower abandonment rate than when not gamified [57, 58]. However, in the present study, the gamified application had an abandonment rate similar to that of the other applications. This could be due to the fact that Pokémon Go was chosen as the gamified application, which may not be the best option for use in the adolescent population, as they perceive it as an old mobile application whose theme does not fit their needs [59]. Therefore, future research should try to analyze whether the use of other gamified mobile applications is better adapted to the interests and needs of adolescents [60], with a positive impact on adherence to the interventions.

The findings allow us to reject the first research hypothesis (H1), as it was expected that overweight/obese adolescents would have a lower adherence to the intervention with mobile physical activity applications and that gamified applications would have a higher adherence than nongamified ones. This is especially important, as mobile applications whose use is promoted from the physical education school subject can motivate overweight/obese adolescents to engage in physical exercise in the same way as normal-weight adolescents.

The second objective of the present investigation was to determine the differences in the objective and subjective assessment of mobile apps according to weight status and mobile app used. The importance of this analysis lies in the fact that previous research has shown the relevance of analyzing the needs that different populations, such as overweight/obese adolescents compared to normal-weight adolescents, may have in the assessment and use of step tracker mobile applications [26]. In this regard, the results showed that there were no significant differences in the assessments of physical activity on mobile apps according to weight status. The scientific literature on the assessment of mobile apps is scarce, especially in the field of physical activity, and only one previous study is known to have shown that the quality of mobile apps was dependent on their functionality and attractiveness [61]. However, the adolescents in the present research who are overweight/obese may have similarly valued the mobile apps as compared to normal-weight adolescents because they may find that the virtual world is a more pleasurable way to engage in physical activity than traditional sports practice [57], in which they may feel harassed or belittled by their peers [62]. These results are similar to those from previous research, in which the use of exergames with adolescents had beneficial results and allowed their use to be maintained over time, regardless of the adolescents' weight status [57].

It is worth noting that in the group of normal-weight adolescents, Pacer obtained better scores in “information” and “usability” as compared to Pokémon Go. This could be due to the fact that Pacer is a pedometer that includes multiple techniques for behaviour change in adolescents [28], which could have favored the use of the mobile application. On the other hand, it is possible that teenagers experienced difficulties with the use of Pokémon Go, as its interface is difficult to use, it requires an internet connection to be used, and it may sometimes fail to accurately determine the location of the device [63]. However, in the overweight/obese adolescent group, there were no significant differences between the apps, suggesting that despite the fact that Pokémon Go may not be age-appropriate, adolescents prefer to exercise with it. Future research should consider the creation of a gamified mobile app adapted to the age of adolescents to encourage physical activity.

The findings obtained allow us to partially accept the second research hypothesis (H2), as significant differences were observed in the assessment made by normal-weight adolescents in some of the mobile applications (Pacer and Pokémon Go), but no significant differences were found in overweight/obese adolescents.

The third objective of the present investigation was to determine the differences in the problematic use of mobile phones according to weight status and the mobile application used by adolescents. Previous research has shown that overweight and obese adolescents, those with a subjective perception of being overweight, and those with inadequate weight control behaviours have higher rates of problematic mobile phone use [18]. However, the results of the present research showed that, after the intervention with mobile applications, neither normal weight nor overweight/obese adolescents showed a higher perception of problematic use of mobile phones. Furthermore, no significant relationships were found between problematic mobile phone use and the adolescents' evaluation of the mobile applications used. Previous studies have shown the problematic use of new technologies in the adolescent population due to the addiction they create in users [15, 16]. In fact, some countries have implemented policies limiting the use of mobile devices in contexts such as education [32], in an attempt to reduce the negative consequences of the addictive use of these devices [16]. However, the results of the present investigation could provide a new dimension to this question, as it cannot be confirmed that adolescents who used mobile applications for physical activity increased their problematic use of mobile phones. These findings could lead to a new line of interventions in which mobile applications are used to promote physical activity in adolescents without the reluctance to use them and without resulting in the addictive use of mobile devices.

Based on the results obtained, the third research hypothesis (H3), which expected an increase in problematic use of the mobile phone after the intervention, can be rejected since neither the group of adolescents with a normal weight nor the overweight/obese group showed an increase in problematic use of the mobile phone after the end of the intervention.

The fourth objective of this research was to establish the relationship between distance travelled with mobile application use, ratings, and problematic mobile phone use. The findings showed a positive relationship between training volume and conflicting mobile phone use in normal-weight adolescents. The results are in line with previous research, suggesting that the use of mobile applications can trigger an increase in mobile phone addiction, which has an impact on the mental health of adolescents [64], even when used for educational purposes [16]. This is because adolescents may be tempted to use other types of mobile applications for recreational purposes, which may divert their attention from physical exercise and affect their well-being [65, 66]. However, it is important to note that the present research did not carry out a linear regression analysis; thus, it cannot be concluded that the use of mobile applications increases conflicting mobile phone use. Therefore, future lines of research should analyze the relationship between these factors.

However, a surprising result of the present investigation is that in overweight/obese adolescents, a relationship between conflictive phone use and training volume was not observed. These results could be explained because previous research found that higher physical activity was associated with less problematic mobile phone use [19, 20]. However, a negative relationship was observed between training volume and perception of mobile applications. In this regard, the adolescents who did not rate the mobile apps well showed a greater engagement in terms of daily steps logged. This also suggests that the incentive provided on the physical education class grade significantly influenced the adherence of overweight/obese adolescents to the intervention, playing an important role in their active participation, as observed in previous research [45, 47]. The mandatory nature of the intervention would make adolescents rate the application worse the more they use it since the initial novelty that is so important for starting to use mobile applications would be lost due to its influence on their motivation [67]. Although Ryan and Deci identified three fundamental psychological needs for the personal development of individuals and the sustainability of intrinsic motivation (competence, autonomy, and social relatedness) [68], González-Cutre et al. added novelty as a fourth influential factor in physical exercise motivation and adherence [69], which could explain the results of the present investigation.

Despite these findings, no correlation was found between increased use of mobile apps and increased problematic mobile phone use in the overweight/obese adolescent population, suggesting that even though they participated in the mandatory intervention, they did not experience negative repercussions in other areas as a result of this participation. This is even more relevant because in normal-weight adolescents, there was a correlation between training volume and problematic mobile phone use. Following the results obtained, the fourth hypothesis (H4) of the present study can be partially accepted. However, it is worth noting that the results showed positive correlations between training volume and problematic mobile phone use in adolescents with normal weight, which partially supports the initial hypothesis; but, in overweight/obese adolescents, a negative correlation was observed between training volume and rating of mobile apps, with no relationship with problematic mobile phone use.

The practical implications of the present study concern the Ministry and Regional Ministry of Education, education centers, and physical education teachers, who could consider the use of mobile applications in physical education classes to promote physical activity in overweight/obese adolescents. This suggests that mobile apps can be an effective tool for increasing physical activity among students, regardless of weight status. The integration of mobile applications as a regular tool in the curriculum of the physical education school subject would make it possible to encourage the practice of physical activity outside school hours as homework for the subject, but this would require its promotion from the physical education school subject as well as exhaustive monitoring by teachers to avoid the problematic use of the mobile phone. The fact that the factors of mobile application used and the weight status of the adolescents do not seem to have an effect on the effectiveness of the intervention opens up a wide range of possibilities for carrying out these interventions at schools.

The present study is not without limitations. First, the problematic use of the mobile phone was determined by means of a questionnaire that provided a subjective assessment of this variable but did not include an objective measurement of the time spent using the mobile device or the applications used. Secondly, the small sample size meant that the allocations of adolescents to each group were small, making it difficult to extrapolate the results to the population of overweight/obese adolescents. And thirdly, also related to the small sample size, it was not possible to perform a linear regression analysis to determine the direction of causality of the correlational analysis performed. Therefore, future research in this area is required to further investigate these aspects.

## 5. Conclusions

In conclusion, the results of the present study indicate that there is a similar adherence between normal weight and overweight/obese adolescents to the intervention, with a similar dropout rate in all of the mobile applications, with no significant differences between them. In addition, it was observed that adolescents, regardless of their weight status, showed a favorable perception towards the use of mobile applications for the promotion of physical activity, especially highlighting the Pacer app, suggesting that these tools can be useful for promoting physical activity from the education center in this population. On the other hand, no significant differences were found in problematic mobile use with different mobile apps according to weight status. However, it should be noted that in the case of normal-weight adolescents, a positive relationship was found between a greater distance travelled with the use of apps and problematic use of the mobile phone, while in overweight/obese adolescents it was not.

## Figures and Tables

**Table 1:** Rate of dropout according to weight status and mobile app used.

**App used**	**Initial sample (** **n** **)**	**Final sample (** **n** **, %)**	**Dropout (** **n** **, %)**	**Adj res. dropout/nondropout**	**Group diff. (** **χ** ^2^, **p)**	**Contingency coefficient**
Total sample according to mobile app used						
Pokémon Go	22	21 (95.45%)	1 (4.55%)	2.1/−2.1	*χ* ^2^ = 4.75 *p* = 0.191	0.226
Strava	22	17 (77.27%)	5 (22.73%)	−0.3/0.3		
Pacer	22	16 (72.73%)	6 (27.27%)	−0.9/0.9		
Map My Walk	22	16 (72.73%)	6 (27.27%)	−0.9/0.9		
Sample divided according to weight status and app used						
Pokémon Go—NW	11	10 (90.91%)	1 (9.09%)	1.0/−1.0	*χ* ^2^ = 9.78 *p* = 0.202	0.316
Pokémon Go—OW/OB	11	11 (100.0%)	0 (0.0%)	1.0/−1.0		
Strava—NW	11	10 (90.91%)	1 (9.09%)	0.2/−0.2		
Strava—OW/OB	11	7 (63.64%)	4 (36.36%)	0.2/−0.2		
Pacer—NW	11	9 (81.82%)	2 (18.18%)	1.8/−1.8		
Pacer—OW/OB	11	7 (63.64%)	4 (36.36%)	−1.4/1.4		
Map My Walk—NW	11	9 (81.82%)	2 (18.18%)	−1.4/1.4		
Map My Walk—OW/OB	11	7 (63.64%)	4 (36.36%)	−1.4/1.4		

**Table 2:** Differences in mobile app rating between normal weight and overweight/obese adolescents.

	**App used**	**Normal weight**	**Overweight/obesity**	**Mean diff. (normal–over)**	**F**	**p**	**95% CI**	**Effect size**
Engagement	Pokémon Go	3.06 ± 1.70	3.36 ± 1.35	−0.30 ± 0.47	0.410	0.524	−1.252; 0.644	0.007
	Strava	3.96 ± 0.58	4.00 ± 0.52	−0.04 ± 0.54	0.006	0.941	−1.109; 1.029	0.000
	Pacer	3.71 ± 0.59	3.63 ± 0.63	0.08 ± 0.55	0.023	0.881	−1.011; 1.176	0.000
	Map My Walk	3.71 ± 0.75	3.31 ± 1.56	0.40 ± 0.55	0.526	0.471	−0.697; 1.490	0.008

Functionality	Pokémon Go	2.88 ± 1.80	3.36 ± 1.31	−0.49 ± 0.49	1.007	0.319	−1.462; 0.485	0.016
	Strava	3.95 ± 0.52	4.46 ± 0.39	−0.51 ± 0.55	0.877	0.353	−1.612; 0.583	0.014
	Pacer	4.11 ± 0.77	4.18 ± 0.43	−0.07 ± 0.56	0.014	0.905	−1.190; 1.055	0.000
	Map My Walk	4.19 ± 0.60	3.96 ± 1.76	0.23 ± 0.56	0.168	0.683	−0.892; 1.353	0.003

Aesthetics	Pokémon Go	2.83 ± 2.04	3.30 ± 1.44	−0.47 ± 0.53	0.789	0.378	−1.527; 0.588	0.013
	Strava	4.07 ± 0.60	4.19 ± 0.47	−0.12 ± 0.60	0.043	0.836	−1.316; 1.069	0.001
	Pacer	3.96 ± 0.77	4.05 ± 0.45	−0.09 ± 0.61	0.019	0.890	−1.304; 1.135	0.000
	Map My Walk	4.07 ± 0.64	3.81 ± 1.76	0.27 ± 0.61	0.188	0.666	−0.955; 1.484	0.003

Information	Pokémon Go	2.58 ± 1.98	3.32 ± 1.60	−0.74 ± 0.55	1.802	0.184	−1.850; 0.364	0.028
	Strava	3.93 ± 0.79	4.39 ± 0.38	−0.47 ± 0.62	0.561	0.457	−1.716; 0.780	0.009
	Pacer	4.19 ± 0.86	4.07 ± 0.37	0.12 ± 0.64	0.037	0.848	−1.154; 1.400	0.001
	Map My Walk	3.89 ± 0.84	3.89 ± 1.82	−0.00 ± 0.64	0.000	0.995	−1.281; 1.273	0.000

Usability	Pokémon Go	2.13 ± 1.70	2.80 ± 1.27	−0.67 ± 0.46	2.087	0.154	−1.598; 0.257	0.033
	Strava	3.23 ± 0.48	3.57 ± 0.62	−0.35 ± 0.52	0.438	0.511	−1.393; 0.700	0.007
	Pacer	3.47 ± 0.71	3.11 ± 0.59	0.37 ± 0.54	0.465	0.498	−0.705; 1.435	0.007
	Map My Walk	3.28 ± 0.67	3.18 ± 1.50	0.10 ± 0.54	0.034	0.854	−0.971; 1.169	0.001

Perceived impact	Pokémon Go	2.17 ± 1.82	2.94 ± 1.38	−0.77 ± 0.52	2.246	0.139	−1.804; 0.258	0.035
	Strava	3.30 ± 0.64	3.45 ± 1.04	−0.15 ± 0.58	0.069	0.794	−1.315; 1.010	0.001
	Pacer	3.67 ± 0.79	3.19 ± 0.74	0.48 ± 0.60	0.641	0.426	−0.713; 1.665	0.010
	Map My Walk	2.87 ± 0.79	3.14 ± 1.53	−0.27 ± 0.60	0.210	0.648	−1.461; 0.916	0.003

**Table 3:** Differences in mobile app rating and problematic use of mobile phones in normal weight and overweight/obesity according to the app used.

	**Weight status**	**Pokémon Go**	**Strava**	**Pacer**	**Map My Walk**	**F**	**p**	**Effect size**
Engagement	Normal weight	3.06 ± 1.70	3.96 ± 0.58	3.71 ± 0.59	3.71 ± 0.75	1.254	0.298	0.057
	Overweight/obesity	3.36 ± 1.35	4.00 ± 0.52	3.63 ± 0.63	3.31 ± 1.56	0.631	0.598	0.030
Functionality	Normal weight	2.88 ± 1.80	3.95 ± 0.52	4.11 ± 0.77	4.19 ± 0.60	2.953	0.059	0.125
	Overweight/obesity	3.36 ± 1.31	4.46 ± 0.39	4.18 ± 0.43	3.96 ± 1.76	1.600	0.198	0.072
Aesthetics	Normal weight	2.83 ± 2.04	4.07 ± 0.60	3.96 ± 0.77	4.07 ± 0.64	2.439	0.073	0.106
	Overweight/obesity	3.30 ± 1.44	4.19 ± 0.47	4.05 ± 0.45	3.81 ± 1.76	0.952	0.421	0.044
Information	Normal weight	2.58 ± 1.98	3.93 ± 0.79	4.19 ± 0.86	3.89 ± 0.84	3.211	0.029	0.134
	Overweight/obesity	3.32 ± 1.60	4.39 ± 0.38	4.07 ± 0.37	3.89 ± 1.82	1.148	0.337	0.053
Usability	Normal weight	2.13 ± 1.70	3.23 ± 0.48	3.47 ± 0.71	3.28 ± 0.67	3.209	0.029	0.134
	Overweight/obesity	2.80 ± 1.27	3.57 ± 0.62	3.11 ± 0.59	3.18 ± 1.50	0.771	0.515	0.036
Perceived impact	Normal weight	2.17 ± 1.82	3.30 ± 0.64	3.67 ± 0.79	2.87 ± 0.79	2.869	0.044	0.122
	Overweight/obesity	2.94 ± 1.38	3.45 ± 1.04	3.19 ± 0.74	3.14 ± 1.53	0.273	0.845	0.013

**Table 4:** Bonferroni's post hoc analysis of the differences in mobile app rating in normal-weight adolescents according to the app used.

	**Weight status**	**App 1**	**App 2**	**Mean diff.**	**p**	**95% CI**
Information	Normal weight	Pokémon Go	Pacer	−1.619 ± 0.582	0.043	−3.206; −0.032
Usability	Normal weight	Pokémon Go	Pacer	−1.347 ± 0.488	0.046	−2.678; −0.017
Perceived impact	Normal weight	Pokémon Go	Pacer	−1.500 ± 0.542	0.045	−2.978; −0.022

**Table 5:** Differences between pre- and posttest in problematic mobile phone use in normal weight and overweight/obese adolescents.

**Descriptors (M ±SD)**								**Covariate objective assessment of the app through uMARS questionnaire**		
	**Mobile app**	**Pre**	**Post**	**Mean diff.**	**p**	**95% CI**	**Effect size**	**p**	**95% CI**	**Effect size**
CERM score										
Normal weight	Pokémon Go	1.80 ± 0.52	1.84 ± 0.78	−0.04 ± 0.15	0.774	−0.341; 0.255	0.001	0.736	−0.352; 0.250	0.002
	Strava	1.44 ± 0.31	1.48 ± 0.47	−0.04 ± 0.12	0.749	−0.289; 0.209	0.002	0.779	−0.287; 0.216	0.001
	Pacer	1.64 ± 0.38	1.63 ± 0.48	0.01 ± 0.14	0.929	−0.266; 0.291	0.000	0.920	−0.266; 0.294	0.000
	Map My Walk	1.60 ± 0.35	1.60 ± 0.56	0.00 ± 0.13	1.000	−0.263; 0.263	0.000	0.974	−0.260; 0.269	0.000
Overweight/obesity	Pokémon Go	1.69 ± 0.52	1.62 ± 0.38	0.07 ± 0.12	0.542	−0.165; 0.310	0.006	0.629	−0.185; 0.304	0.004
	Strava	1.41 ± 0.29	1.44 ± 0.17	−0.03 ± 0.15	0.848	−0.326; 0.269	0.001	0.917	−0.319; 0.287	0.000
	Pacer	1.61 ± 0.30	1.74 ± 0.61	−0.13 ± 0.15	0.391	−0.426; 0.169	0.013	0.412	−0.424; 0.176	0.012
	Map My Walk	1.41 ± 0.23	1.47 ± 0.22	−0.06 ± 0.15	0.702	−0.355; 0.241	0.003	0.694	−0.359; 0.241	0.003
Conflictive use										
Normal weight	Pokémon Go	1.38 ± 0.70	1.28 ± 0.99	0.10 ± 0.17	0.553	−0.235; 0.435	0.006	0.855	−0.368; 0.306	0.001
	Strava	1.32 ± 0.42	1.26 ± 0.56	0.06 ± 0.17	0.722	−0.275; 0.395	0.002	0.546	−0.225; 0.420	0.006
	Pacer	1.24 ± 0.22	1.31 ± 0.39	−0.07 ± 0.18	0.707	−0.420; 0.286	0.002	0.879	−0.366; 0.314	0.000
	Map My Walk	1.29 ± 0.35	1.47 ± 0.81	−0.18 ± 0.18	0.318	−0.531; 0.175	0.016	0.409	−0.481; 0.199	0.011
Overweight/obesity	Pokémon Go	1.33 ± 0.52	1.29 ± 0.41	0.04 ± 0.16	0.821	−0.283; 0.356	0.001	0.896	−0.330; 0.289	0.000
	Strava	1.20 ± 0.28	1.23 ± 0.21	−0.03 ± 0.20	0.887	−0.429; 0.372	0.000	0.791	−0.337; 0.441	0.001
	Pacer	1.31 ± 0.34	1.46 ± 0.55	−0.14 ± 0.20	0.478	−0.543; 0.258	0.008	0.591	−0.489; 0.281	0.005
	Map My Walk	1.20 ± 0.16	1.20 ± 0.26	0.00 ± 0.20	1.000	−0.400; 0.400	0.000	0.985	−0.380; 0.388	0.000
Emotional use										
Normal weight	Pokémon Go	1.88 ± 0.85	1.52 ± 1.04	0.36 ± 0.19	0.058	−0.013; 0.733	0.057	0.311	−0.180; 0.555	0.017
	Strava	1.56 ± 0.35	1.70 ± 0.47	−0.14 ± 0.19	0.456	−0.513; 0.233	0.009	0.610	−0.441; 0.261	0.004
	Pacer	1.91 ± 0.70	1.82 ± 0.68	0.09 ± 0.20	0.653	−0.304; 0.482	0.003	0.445	−0.228; 0.513	0.010
	Map My Walk	1.91 ± 0.48	1.73 ± 0.39	0.18 ± 0.20	0.370	−0.215; 0.571	0.013	0.227	−0.144; 0.596	0.024
Overweight/obesity	Pokémon Go	2.05 ± 0.61	1.95 ± 0.47	0.11 ± 0.18	0.542	−0.247; 0.465	0.006	0.839	−0.303; 0.371	0.001
	Strava	1.63 ± 0.34	1.66 ± 0.19	−0.03 ± 0.22	0.898	−0.474; 0.417	0.000	0.717	−0.347; 0.501	0.002
	Pacer	1.91 ± 0.45	2.03 ± 0.72	−0.11 ± 0.22	0.610	−0.560; 0.332	0.004	0.764	−0.483; 0.356	0.001
	Map My Walk	1.63 ± 0.34	1.74 ± 0.38	−0.11 ± 0.22	0.610	−0.560; 0.332	0.004	0.603	−0.527; 0.309	0.004

**Table 6:** Correlation analysis between the steps taken with the use of the app and the app evaluation in normal weight and overweight/obese adolescents.

	**Training volume**	**Engagement**	**Functionality**	**Aesthetics**	**Information**	**Usability**	**Perceived impact**	**Conflictive use**	**Emotional use**
Normal weight									
Training volume	—	—	—	—	—	—	—	—	—
Engagement	−0.115; *p* = 0.493	—	—	—	—	—	—	—	—
Functionality	−0.128; *p* = 0.444	0.874; *p* < 0.001	—	—	—	—	—	—	—
Aesthetics	−0.260; *p* = 0.115	0.841; *p* < 0.001	0.882; *p* < 0.001	—	—	—	—	—	—
Information	−0.150; *p* = 0.368	0.787; *p* < 0.001	0.861; *p* < 0.001	0.922; *p* < 0.001	—	—	—	—	—
Usability	−0.145; *p* = 0.385	0.773; *p* < 0.001	0.849; *p* < 0.001	0.844; *p* < 0.001	0.925; *p* < 0.001	—	—	—	—
Perceived impact	−0.148; *p* = 0.376	0.734; *p* < 0.001	0.811; *p* < 0.001	0.781; *p* < 0.001	0.846; *p* < 0.001	0.864; *p* < 0.001	—	—	—
Conflictive use	0.445; *p* = 0.005	0.480; *p* = 0.002	0.400; *p* = 0.013	0.274; *p* = 0.095	0.259; *p* = 0.116	0.378; *p* = 0.019	0.246; *p* = 0.137	—	—
Emotional use	0.189; *p* = 0.256	0.506; *p* = 0.001	0.413; *p* = 0.010	0.303; *p* = 0.064	0.266; *p* = 0.106	0.385; *p* = 0.017	0.218; *p* = 0.188	0.750; *p* < 0.001	—
Overweight/obesity									
Training volume	—	—	—	—	—	—	—	—	—
Engagement	−0.455; *p* = 0.009	—	—	—	—	—	—	—	—
Functionality	−0.504; *p* = 0.003	0.859; *p* < 0.001	—	—	—	—	—	—	—
Aesthetics	−0.475; *p* = 0.006	0.913; *p* < 0.001	0.907; *p* < 0.001	—	—	—	—	—	—
Information	−0.418; *p* = 0.017	0.861; *p* < 0.001	0.923; *p* < 0.001	0.935; *p* < 0.001	—	—	—	—	—
Usability	−0.395; *p* = 0.025	0.817; *p* < 0.001	0.867; *p* < 0.001	0.885; *p* < 0.001	0.903; *p* < 0.001	—	—	—	—
Perceived impact	−0.473; *p* = 0.006	0.710; *p* < 0.001	0.833; *p* < 0.001	0.786; *p* < 0.001	0.816; *p* < 0.001	0.817; *p* < 0.001	—	—	—
Conflictive use	0.007; *p* = 0.971	−0.110; *p* = 0.549	0.061; *p* = 0.739	−0.073; *p* = 0.692	0.037; *p* = 0.840	0.008; *p* = 0.967	0.141; *p* = 0.442	—	—
Emotional use	0.057; *p* = 0.757	−0.065; *p* = 0.724	0.118; *p* = 0.521	−0.022; *p* = 0.907	0.011; *p* = 0.951	0.018; *p* = 0.922	0.157; *p* = 0.389	0.592; *p* < 0.001	—

## Data Availability

The data that support the findings of this study are available from the corresponding author upon reasonable request.
